# Willing to Be Involved in Cancer

**DOI:** 10.3390/genes7070037

**Published:** 2016-07-18

**Authors:** Frank J. Gunn-Moore, Andrew M. Tilston-Lünel, Paul A. Reynolds

**Affiliations:** 1 Medical and Biological Sciences Building, School of Biology, University of St Andrews, St Andrews KY16 9TF, UK; aml7@st-andrews.ac.uk; 2 Medical and Biological Sciences Building, School of Medicine, University of St Andrews, St Andrews KY16 9TF, UK

**Keywords:** willin, FRMD6, FERM proteins, cancer

## Abstract

Genome sequencing is now a common procedure, but prior to this, screening experiments using protein baits was one of the routinely used methods that, occasionally, allowed the identification of new gene products. One such experiment uncovered the gene product called willin/human Expanded/FRMD6. Initial characterization studies found that willin bound phospholipids and was strongly co-localised with actin. However, subsequently, willin was found to be the closest human sequence homologue of the *Drosophila* protein Expanded (Ex), sharing 60% homology with the Ex FERM domain. This in turn suggested, and then was proven that willin could activate the Hippo signalling pathway. This review describes the increasing body of knowledge about the actions of willin in a number of cellular functions related to cancer. However, like many gene products involved in aspects of cell signalling, a convincing direct role for willin in cancer remains tantalisingly elusive, at present.

## 1. History

Before the sequencing of the human genome was completed, biochemical screening experiments had the extra bonus of occasionally uncovering new genes. These moments of serendipity are the great ally for a scientist and such a moment played an important part in the identification of the gene product called willin/human Expanded/FRMD6.

Previously, FJG-M was involved in the identification of the protein complex that attaches myelin to nerve cells in the mammalian nervous system. The work resulted in the identification of the 155 kDa isoform of neurofascin as the transmembrane receptor that bound the paranodal loops of myelinating glia (both oligodendrocytes and Schwann cells) to the CASPR (contactin-associated-protein) and contactin receptors of the neuronal surface (subsequently published in [1,2]). However, how did the 155 kDa neurofascin receptor interact with the intracellular pathways of these cells? Previous experiments had identified ankyrin, thus linking this receptor to the cytoskeleton [3], but the short intracellular domain of neurofascin had other recognisable protein motifs. The cytoplasmic tail of neurofascin was duly screened for its potential binding partners using a bespoke yeast-two- hybrid library, thereby identifying potentially novel interacting proteins.

One such identified protein was ezrin, which was subsequently shown to bind to neurofascin’s cytoplasmic domain in what was identified as a new sub-set of FERM (4.1, ezrin, radixin, moesin) binding motifs, and more importantly was shown to physiologically occur at the location where Schwann cells interdigitate above the Node of Ranvier [4]. A second protein initially termed 163ScII had a recognisable FERM domain, but the sequence itself was new, and so even though this was prior to the human genome project, it was known to be a new gene. Further data mining, and cloning of the human, mouse and rat forms led to identification of the full-length gene, which was still without a name. As the gene had obvious similarities to ezrin, which itself had been named after a person (but had also been named “cytovillin” or “villin-2” [5,6,7]), the name “willin” was coined as the work was being performed in the “William Dick” Veterinary School, University of Edinburgh [8].

## 2. Willin, the Protein

**Figure 1:**
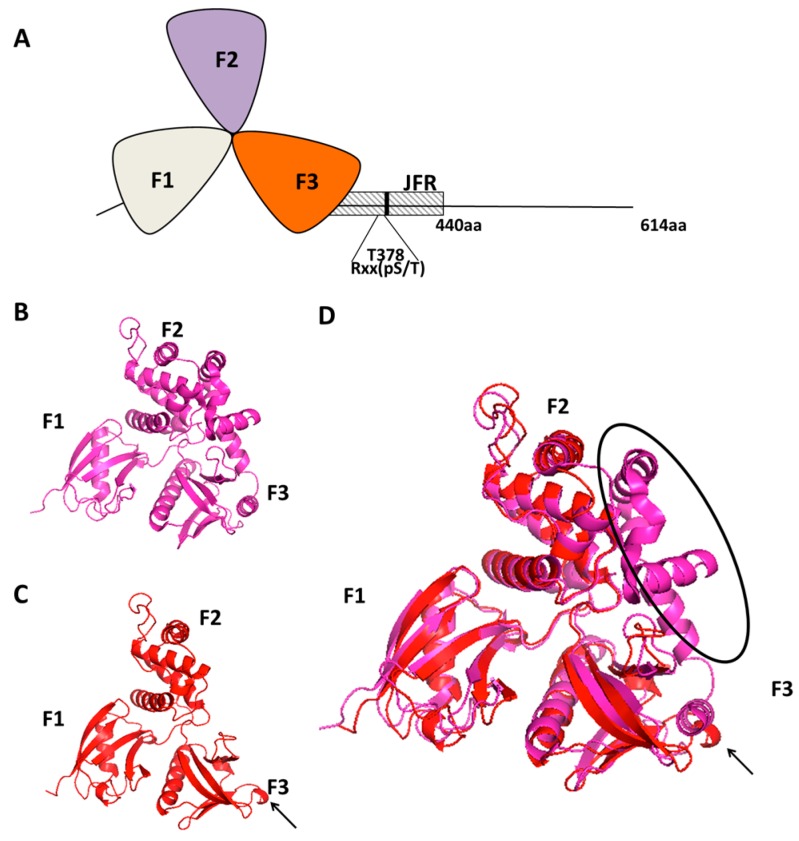
(**A**) A schematic representation of the *N*-terminal clover leaf shaped FERM domain of Willin (containing the F1, F2 and F3 lobes 30–330 aa) and a JFR (juxta-FERM domain region 330–440 aa) which contains a14-3-3 recognition consensus sequence (Rxx(pS/T)); (**B**) The Crystal structure of the FERM domain of Ezrin 4RM9; (**C**) The predicted crystal structure of the FERM domain of willin using Phyre 2; (**D**) The predicted three-dimensional structure of the FERM domain of willin is superimposed over the crystal structure of the FERM domain of Ezrin 4RM9. The classical cloverleaf structure can be seen in the predicted FERM domain of willin. Black arrow points to an additional β-sheet on the F3 lobe of willin’s FERM domain. Black elliptical region highlights the extra α-helices that extend from the F3 lobe to shield the F2 lobe of ezrin.

Initially, the FERM domain of willin was the most recognisable protein motif and had been the initial evidence that this new sequence encoded for a protein. The FERM domain is a slightly unusual protein motif as it has the capability of binding both proteins and phospholipids (reviewed in [9]). Previously, FJG-M had identified other phospholipid binding proteins and shown that the cellular distribution of proteins such as GRP-1 and cytohesin-1 were influenced by growth factors [10,11]. Therefore, the first work on willin, involved investigating some of the basic biochemical parameters of this protein. The FERM domain of willin is in the *N*-terminus as occurs with the ERM proteins and merlin, and though it only had up to 47% homology, the FERM domain of willin retains the capability of folding into a recognisable FERM structure (Figure 1A) [9]. A comparison of the structure of Ezrin’s FERM domain along with the predicted FERM domain of Willin shows the similarity and differences between the two FERM domains (Figure 1B–D). More directly, it could bind phospholipids, and the intracellular distribution could be influenced by the addition of growth factors, though intriguingly this did not require the activity of phosphoinositide 3-kinase which normally would be predicted to be involved [8]. Willin intracellular distribution also appeared to be influenced by cell-cell contact, and indeed in this manner it strongly resembled radixin, which had been previously shown to be associated within cleavage furrows [12]. In addition, willin had a potential linkage to the cytoskeleton, as it was strongly co-localised with actin, though no direct binding was thought to occur as no direct sequences could be identified (as occurs in ezrin), but the addition of cytochalasin D had no effect on willin distribution [8]. Additionally, green fluorescent protein (GFP) tagged willin showed occasional nuclear localization, although the significance of this was unknown, and at first was thought to be due to supra-physiological expression, or cleavage of the GFP tag [13].

It was after this initial work that willin’s possible tentative linkage to cancer was first explored. Collaborators had identified that the cytoplasmic distribution of ezrin could predict the outcome of particular head and neck cancer studies, and as such act as a prognostic marker for these tumours [14]. Within this first study, willin (as it was a new member of the FERM family, and potentially could influence other FERM proteins [15]) and moesin expression and their intracellular distribution, were additionally studied in these squamous cell carcinomas. With respect to willin, its mixed intracellular distribution was confirmed: endogenous willin was found in the nucleus, cytoplasm or plasma membrane, and this appeared to depend on the location of the cells within the tissue. In a follow on study, it was also noted that the distribution of ezrin might be influenced by moesin, willin or merlin, as the latter were expressed differently in the different tissues layers. The fact that the ERM proteins had previously been shown to bind both as homo- and hetero-dimers was not lost on the authors [15]. Therefore, initial studies implied a circumstantial involvement of willin, which may be acting through other known proteins.

## 3. Willin and Signalling Pathways

It was at this time that a publication on *Drosophila’s* expanded provided a potential connection between willin and the Hippo signalling pathway [16]. Specifically, in the appendix of this paper on the role of merlin and expanded controlling proliferation and apoptosis via the newly emerging Hippo pathway, was reported a DNA sequence that was reported to be the human equivalent of expanded (hEx). Serendipity struck again as this sequence was identical to willin. The author PAR, who had previously worked on signal transduction pathways involved in cancer [17,18], heard a talk on the potential link of willin with a rapidly emerging new signalling pathway, and so we decided to pursue the question does willin/hEx activate the Hippo signalling pathway?

### 3.1. Hippo Pathway

The Salvador/Warts/Hippo (Hippo) signalling pathway defines a signalling cascade regulating a number of processes important in cell growth and proliferation in mammals, including cell contact inhibition, organ size control and apoptosis. The Hippo pathway core kinase cassette consists of a series of kinases and adaptor proteins that restrain the activity of a nuclear effector, and so is a very unusual kinase pathway. A complex of the Hippo kinase (Hpo) and an adaptor protein Salvador, phosphorylates and activates Warts kinase (Wts) and an activating subunit, Mats [19,20,21]. This core kinase cassette phosphorylates and inactivates the transcriptional co-activator Yorkie (Yki), thereby suppressing gene expression. Phenotypically, inactivation of Yki in *Drosophila* imaginal disc tissues results in cell cycle arrest and apoptosis [19,20,21]. A tripartite complex consisting of Ex, Mer and Kibra function upstream of the core kinase cassette, activating the pathway via Hpo and Wts phosphorylation [16,22,23]. Additionally, Ex forms a complex with Yki and is proposed to directly regulate its activity by the WW domains of Yki and the PPXY motifs of Ex [24]. Interestingly, Ex has been implicated in the proper regulation of growth, as demonstrated by the observed overgrowth phenotypes in adult wings and larval wing imaginal disc tissue of Ex deficient *Drosophila* [25]. The interaction between the FERM proteins Ex and Mer is facilitated by the Ex FERM domain and the C-terminal domain of Mer [26], supporting the previously published data on the occurrence of head-to-tail FERM protein heterodimers [27]. Although Ex and Mer interact and colocalize with each other at the plasma membrane and in the cytoplasm, they individually have been demonstrated to carry out different functions as well: the cell cycle is regulated by Ex whereas Mer has been implicated in apoptosis [26,28].

### 3.2. Ex Is Not Fully Conserved in Mammalian Willin/FRMD6

The Hippo pathway is an evolutionary growth regulatory pathway that has been conserved from flies to mammals: The core components include MST1/2 (Hpo orthologues), WW45/Sav (Sav orthologue), LATS1/2 (Wts orthologues), MOB1 (Mats orthologue) and YAP/TAZ (Yki orthologue) [19]. Despite of having shared core components, the Hippo pathway is activated by upstream components, which exhibit mechanistic differences in flies and mammals [19,29]. Two vertebrate homologues of Ex, Ex1 and Ex2, were reported and as mentioned above the DNA sequence of human Ex1 is identical with willin [8,16], though by this stage HUGO nomenclature had termed willin/hEx as being called FRMD6. On further analysis, willin/FRMD6 shared 60% homology with the FERM domain of Ex, making it the closest human sequence homologue of Ex. Although willin was able to localize in the same compartments as Ex, we showed that it was not able to rescue the overgrowth phenotype induced by the loss of Ex in *Drosophila* [30]. Interestingly, the C-terminal region of Ex shows no similarity to willin, indeed Ex is 1429 amino acids long, as compared with 614 amino acids for willin [8,16]. Therefore, we have previously hypothesised that Ex’s function might be replaced by two proteins in mammals that of willin and AMOTp130, as the latter contains two PPXY motifs that can bind YAP, and as such phenocopy the c-terminal domain of Ex [9].

The differences between Ex and willin are one illustration of the fundamental differences that exist in the upstream regulatory mechanisms of Hippo signalling between *Drosophila* and mammals. An evolutionary shift occurred in the regulation of the Hippo pathway between mammals and flies. As mentioned above, in *Drosophila*, Ex, Mer and Kibra all form a complex that regulates the Hippo pathway [22,23,31]. However, in mammals, ours and others’ data would argue against this possibility and instead suggest that KIBRA acts directly on LATS1/2 in an MST-independent manner [32,33,34]. Echinoid(Ed), an arthropod specific protein, plays a role in Hippo signalling and the formation of adherens junctions [35]. Further evolutionary diversification of Hippo pathway components can be observed by phylogenetic lineage tracing revealing that the Hippo pathway-interacting motif (HM) of Fat, the C-terminal of Ex and Ed are present only in arthropods, AMOT is absent from Neodipterans, and the PDZ-binding motif (PBM) of Yki is absent from Dipterans [36].

We also reported that the *N*-terminal FERM domain of willin is sufficient to activate the Hippo pathway via MST1/2 and can antagonize YAP-induced phenotypes in mammalian cells [30]. Willin activates the Hippo pathway, inducing the phosphorylation of MST1/2, LATS1 and YAP in MCF10A, HEK (human embryonic kidney)-293T cells and primary sciatic nerve fibroblasts [30,37]. Knockdown of willin phenocopied YAP overexpression, inducing phenotypes consistent with epithelial-mesenchymal transition (EMT) in MCF10A cells, and knockdown of willin decreased phosphorylation of MST1/2, LATS1 and YAP [30]. Surprisingly, a recent article described observations in breast cancer MDA-MB-231 cells where no increased phosphorylation of the core kinase cassette was noted when willin was overexpressed in these cells. The authors concluded from this that willin acts independently of the Hippo pathway. A possible explanation for this difference is that high cell confluency may have confounded their findings, masking any phosphorylation increases due to high initial levels. Unfortunately, phosphorylation of the core kinase cassette upon willin knockdown in this context was not investigated [38]. The authors did explore the relationship between willin expression and drug resistance and showed that increased willin expression correlated with increased sensitivity of the MDA-MB-231 cells to the chemotherapeutic drug taxol [38].

### 3.3. Willin Associates with Junctional Components

Further insight into the factors that contribute to willin’s control of the Hippo pathway and junction formation are slowly being uncovered. Recently, willin has been identified to interact with apical polarity components Par3 and aPKC, and these interactions were FERM domain independent, with the authors demonstrating that the region between 330–440 aa [termed JFR (juxta-FERM domain region)] of willin was responsible for this interaction [39]. aPKC was recruited to the plasma membrane by the overexpression of willin in MDCK cells and remained associated to the plasma membrane despite depletion of Par3, demonstrating that both Willin and Par3 were required for aPKC membrane association [39]. This study highlights that although willin lacks a kinase domain, it may be able to affect the phosphorylation of aPKC targets by its interaction with aPKC [39]. In a fibroblast cell line, the association of willin with AJs is dependent on the nectin1-afadin complex [40]. Willin has also been suggested to interact with other junctional components such as RASSF8 however this has been observed in high throughput protein-protein interaction screens such as BioPlex (biophysical interactions of ORFeome-based complexes) performed by the Gygi laboratory and a high throughput Y2H screen performed by the Vidal lab [41,42]. Although there is evidence that RASSF8 and willin can influence adherens junction formation on their own, no research has been conducted in to the physiological role of their interaction [39].

In *Drosophila*, Crb recruits Ex to the plasma membrane via the FERM domain of Ex [43,44,45]. Loss of Crb or mutations within its FBM (FERM-binding motif) results in mislocalization of Ex [43,44,45]. Additionally, misexpression of Crb causes loss of Ex expression, which then leads to disruption of the associated Hippo pathway complexes [23,24]. Intracellular localization of Mer or the transmembrane protein Fat did not change in Crb mutant cells [43,46,47,48,49]. Surprisingly, we have recently shown that in mammalian cells, Crumbs3 binds to the FERM domain of ezrin, rather than the mammalian Ex homologue, willin [50].

### 3.4. Connections to the Actin Cytoskeleton

The dynamic arrangement and behaviour of actin filaments determines the shape of migrating cells, supplying the protrusive force for cell movement and providing the cortical tension necessary for maintaining intercellular and extracellular contacts [51]. The actin cytoskeleton plays important roles by defining structure within a cell and the tissue, but this cytoskeletal component can also function as an intermediator component between the extracellular environment by acting as a mechanosensor which can affect various signalling pathways. For example, the actin cytoskeleton of fibroblasts has been shown to behave differently on substrates with altering stiffness [52]. Importantly, the F-actin cytoskeleton has been shown to play a role in the regulation of various signalling pathways, including the Hippo pathway [51]. Of particular interest is the relationship between the activity of MST2 and the state of the F-actin cytoskeleton. Previously published data demonstrated that the presence of a functional actin cytoskeleton was able to recruit MST1/2 to the cytoskeleton. Subsequent disruption of the cytoskeleton with Cytochalasin D released MST2 resulting in the activation of JNK signalling [53]. Although the Hippo pathway was not investigated during the release of MST2 from the cytoskeleton, it is plausible to consider that this released MST2 might be able to act a switch between ERK signalling and Hippo signalling [54]. Work performed in *Drosophila* has demonstrated that the Capping proteins (CP) act as a negative regulator of the Hippo pathway [55], supporting previously published literature documenting the overgrowth phenotypes observed by the loss of CPs [56]. Similarly, the effects of the loss of CPs can be phenocopied by the induction of actin polymerization by the overexpression of active Diaphanous (an actin nucleation factor) also drives proliferation via the Hippo pathway [56].

**Figure 2:**
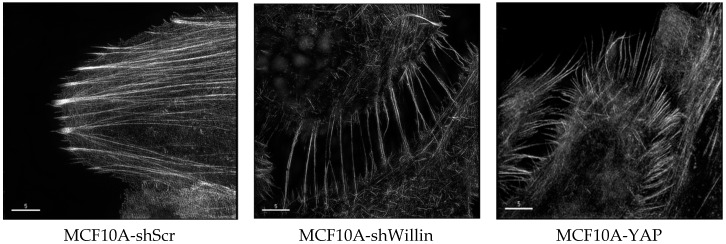
Structured illumination microscope images of Fluor568 phallodin stained actin cytoskeleton of MCF10A cells expressing either shScr (scramble control), shWillin or YAP, indicating that a knockdown of willin expression appears to phenocopy increased YAP-induced filopodia formation [63]. Images taken by Dr S Moleirinho and Dr M Posch (University of Dundee) on an OMX microscope (scale bar, 5 μm).

The actin cytoskeleton is a very dynamic structure that can respond to the extracellular environment that the cells are in contact with, demonstrated by their ability to modulate the intercellular tension accordingly [52,57]. The activity of Hippo pathway components YAP and TAZ have been associated with cytoskeletal tension [58,59]. In order to mimic different physiologically environments, hydrogel matrices with varied degrees of stiffness can be used to culture cells in vitro thereby allowing the cells to mechanosense the environment and modulate the localization of YAP and therefore the activity of YAP. The localization of YAP is cell density dependent, and under high cell density, YAP is predominantly cytoplasmic [60,61]. However, when cells are cultured at high density on an elastic substrate that is subjected to stretching forces, the nuclear localization of YAP is observed [62]. We have unpublished evidence suggesting that willin is able to influence the actin cytoskeleton. The F-actin expression pattern in MCF10A-YAP-Willin and MCF10A-YAP-vector cells, as assessed by phalloidin staining and probed by OMX three-dimensional structured illumination microscopy (3D-SIM), was notably different between the two cell lines. Specifically, expression of willin antagonized the internal cortical actin pattern observed in MCF10A-YAP-vector cells. Importantly, MCF10A-YAP(S127A) cell lines displayed loss of adherens junction’s at cortical sites adjacent to cell-to-cell interface and willin expression did not promote any changes in these phenotypes. The morphological changes were also observed in MCF10A-shWillin cells, when compared to MCF10A-shScr cells, such that MCF10A-shWillin cells presented a dramatic increase in stress fibers with pronounced loss of internal cortical actin organization (Figure 2; ref. [63]). Collectively, these data indicate that willin is able to modify the F-actin cellular organization.

## 4. Willin and Cancer

The involvement of the Hippo pathway, junctional components and the cytoskeleton in cancer is well known and reported. Amplification and overexpression of the Hippo pathway effector, YAP, has been reported in various human and murine tumours. Additionally, a number of hallmarks of cancer including cell proliferation, cell survival and maintenance of a stem-like phenotype have all been associated with various defects in the Hippo pathway [20]. The perturbation of the Hippo pathway in cell culture and in vivo studies results in characteristic phenotypes such as increased cell proliferation, migration, invasion and anti-apoptotic behaviour, which are often observed in premalignant and malignant tissues. Various human carcinomas such as lung, ovarian, liver and prostate cancer have been shown to have aberrant regulation of Hippo pathway components [20]. Although the high frequency of misregulated Hippo components have been reported in these studies, there is very little evidence of germline or somatic mutations within these genes, unlike other signalling pathways involved in cancer progression. However, there are a few rare instances of observed mutations, e.g., *NF2* mutations in schwannomas [20]. An important cellular function that is directly affected in cancer is cell-cell adhesion.

Cell junctions are protein dense regions that play an extremely important role in epithelial tissues, such as the maintenance of apicobasal polarity, intercellular communication, promotion of adhesive properties, compartmentalization of proteins and lipids, and also modulate various signalling cascades. The dysregulation of cellular adhesion plays a critical role in the process of malignant transformation and metastasis and defects in junction genes have been widely reported in breast, prostate, ovarian, endometrial, lung, liver and colorectal carcinomas [64]. Disruption of factors contributing to adherens junctions (AJs), such as E-cadherin occurs in processes associated with cancer, such as cadherin switching during epithelial-mesenchymal transition (EMT) [33,64]. Tight junctions (TJs) are the most apical of junctions, and compromised of effector molecules (e.g., CRB3), scaffold proteins (e.g., ZO-1) and the transmembrane proteins such as JAMs, occludin and claudins [65]. TJs functions include the barrier function that regulates the permeability of monolayers, and the fence function that maintains the lipid and protein composition of the apical and basal plasma membranes and promotes structure to cells and integrity to the tissue. The disruption of cellular junctions is observed during tumour progression that results the impairment to tissue organization and barrier functions of epithelial layers [66,67,68,69].

The tight balance between proliferation and apoptosis, the regulation of functional cellular junctions, a stable and well functioning actin cytoskeleton, along with tightly regulated transcriptional control in all cells is collectively necessary to maintain epithelial tissue homeostasis. The loss of these epithelial characteristics often begins at the onset of cancer. Once these cells also have lost their epithelial characteristics and are undergoing an epithelial-mesenchymal transition (EMT) shift, they need to be able to reorganize their actin cytoskeleton to be able exhibit directional cell migration and cell elongation to have increased motility [67,70,71,72]. Due to the dynamic properties of the actin cytoskeleton, cancer cells have the ability to modulate their signalling, adhesion and mechanical properties to be able to survive in their microenvironment resulting in tumour progression and increased metastatic potential, although currently there is no definitive role of actin bundling in metastasis [73].

Since willin appears to effect all of these cellular functions, this leads to the question is willin involved in cancer? While willin might act as a tumour suppressor, like merlin, only a background frequency of willin mutations have been reported in cancer at present (http://cancer.sanger.ac.uk/cosmic/gene/analysis?ln=FRMD6), so it is difficult to argue that these are driver mutations in cancer. However, it is at the protein level that involvement of a gene product in human disease is proved, so what do we know? As mentioned above, our collaborators had initially shown that intracellular distribution of proteins can act as a marker or predictor for development of certain cancers, though ezrin’s cytoplasmic expression proved to be significant, willin’s distribution did not [14]. In a follow up study, 131 samples from head and neck cancer patients were further evaluated for ezrin, moesin, merlin and willin, recording levels of expression and also cellular distribution of cytoplasmic, membranous or nuclear [15]. All of these four different FERM proteins again showed expression in different parts of the cell. However, poor clinical outcome was only correlated with high levels of cytoplasmic ezrin, whereas the intensity of cytoplasmic staining for willin, merlin or moesin did not play a role in determining clinical outcome. Although increased nuclear localization of willin and merlin could predict clinical outcome, further evidence is needed to determine this observation in disease progression [15]. In addition, it is noteworthy that the increase in cytoplasmic ezrin expression resulted in changes of numerous gene transcripts some of which are linked to the Hippo pathway and cytoskeleton [15].

We would predict that willin’s involvement in cancer appears to be one from indirect effects, but also the fact that the FERM proteins have a history of being involved in cancer. For example, merlin/NF2 is a complex gene that is involved in a number of signalling pathways including Src/Fak, Hippo, Ras/Rac/PAK, ERK1/2, AKT and CRL4-DCAF, with somatic *NF2* mutations occurring in various cancers [74]. Focal adhesion kinase (FAK) expression is elevated in many human cancers and a FERM domain-mediated FAK cell survival pathway involving p53 may be functional in tumour cells [75]. Ezrin, radixin and moesin not only serve as linker molecules between the actin cytoskeleton and the plasma membrane, but also have been shown to play a role in the generation of microvilli and contribute to proper epithelial morphogenesis [76]. Clinical studies have implicated the expression levels and cytoplasmic localization of ezrin in breast cancer, colorectal cancer, lung cancer, prostate cancer, uterine cervical cancer, endometrioid carcinomas, and pancreatic ductal adenocarcinomas and may predict poor clinical outcome [15,76,77,78,79]. Although evidence is lacking of willin mutations in cancer, a tumour suppressor role for willin is suggested by ezrin’s antagonistic effects on willin in its ability to phosphorylate MST1/2 (ref. [30]). In a wider context, ezrin may counteract the effects of willin on the Hippo pathway. Indeed many of our previous studies have shown that willin can antagonise the effect of YAP, and a decrease in willin expression can phenocopy an increase in YAP expression [30]. At the biochemical level this could be due to the way the FERM proteins can form both homo-dimers and heterodimers [80]. Indeed, we have previously identified a protein complex containing willin, ezrin and merlin [81]. Also the fact that these interactions are controlled by FERM domains would fit with our finding that only the FERM domain of willin was necessary to lead to the activation of the hippo pathway [30]. Interestingly, as well as merlin, ezrin and willin, we have reported that there appears to be a series of other newly identified FERM containing proteins which can connect the hippo pathway, junctional components and the cytoskeleton [9] all of which can influence cancer.

**Figure 3:**
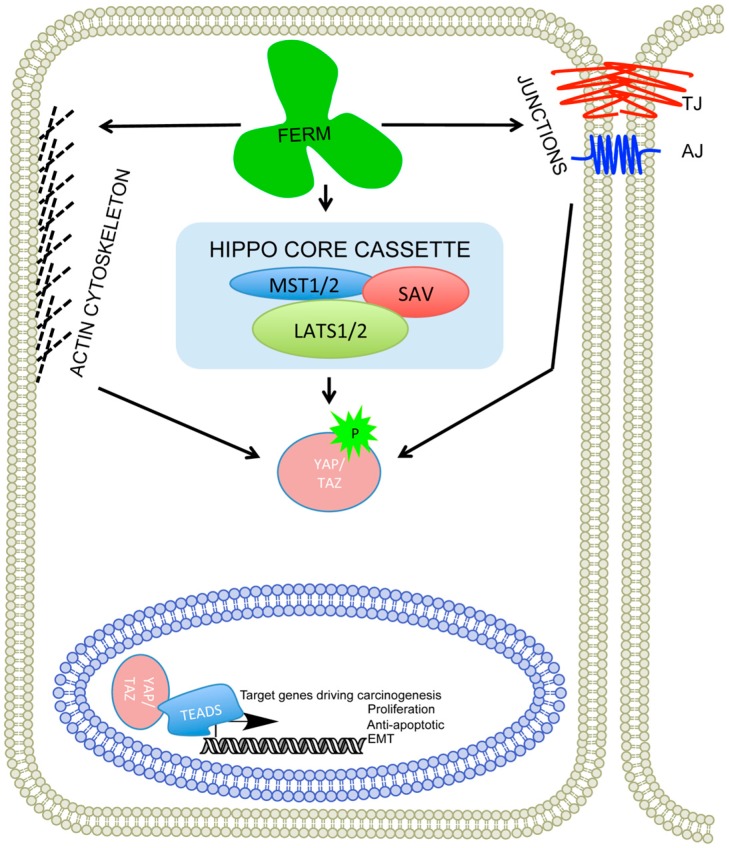
Willin, merlin and ezrin are all FERM containing proteins that can influence proper functioning of the cytoskeleton, cell to cell junction complexes (TJ: tight junctions, AJ: adherens junctions) and the hippo signalling pathway. The perturbation of the FERM proteins result in nuclear localization of WW proteins (YAP/TAZ) switching on the transcription of target genes, many of which are involved in cancer progression.

Therefore, we would predict that just as the Hippo pathway is defined by the WW domain, there may be a controlling complex of proteins that are defined by their FERM domains and willin/hEx/FRMD6 acts as one of these proteins (Figure 3). It would seem fitting that the FERM domain that provided the first serendipitous observation of a new protein, may have a defining role in development and the control of cancer.

## Abbreviations

The following abbreviations are used in this manuscript:

3D-SIM: three-dimensional structured illumination microscopy
aPKC: atypical Protein Kinase C
AJ: adherens junction
CASPR: contactin associated protein
Ed: Echinoid
Ex: Expanded
EMT: epithelial-mesenchymal transition
FAK: focal adhesion kinase
FERM: Four point one, ezrin, radixin, moesin
HEK: human embryonic kidney
HM: Hippo pathway-interacting motif
TJ: tight junction
Y2H: yeast two hybrid
